# A multi‐scale analysis of basketball throw in virtual reality for tracking perceptual‐motor expertise

**DOI:** 10.1111/sms.14250

**Published:** 2022-11-18

**Authors:** Pooya Soltani, Antoine H. P. Morice

**Affiliations:** ^1^ School of Digital, Technologies and Arts Staffordshire University Stoke‐on‐Trent UK; ^2^ Centre for the Analysis of Motion, Entertainment Research and Applications (CAMERA), Department of Computer Science, Department for Health University of Bath Bath UK; ^3^ Aix‐Marseille University, CNRS, ISM Marseille France

**Keywords:** basketball shooting, distance perception, gender, sports simulator, validation

## Abstract

To benefit from virtual reality (VR) as a complementary tool for training, coaches must determine the proper tools and variables for tracking sports performance. We explored the basketball shooting at several scales (basket‐ball, ball‐player, and player systems) by monitoring success‐rate, and ball and body kinematics. We measured how these scales of analysis allowed tracking players' expertise and perceptual sensitivity to basket distance. Experienced and novice players were instructed to naturally throw and swish an instrumented ball in a stereoscopically rendered virtual basket. We challenged their perceptual‐motor systems by manipulating the distance of the virtual basket while keeping the surrounding environment unchanged. The success‐rate accounted for the players' shooting adjustments to the manipulation of basket distance and allowed tracking their expertise. Ball kinematics also reflected the manipulation of distance and allowed detecting gender, but did not reflect the players' expertise. Finally, body kinematics variables did not echo players' adjustments to the distance manipulation but reflected their expertise and gender. The results gained at each scale of analysis are discussed with regard to the simulator's construct, biomechanical, and psychological fidelity.

## 1 INTRODUCTION

What can a virtual reality (VR) setup reveal about sports performance? This question may arise for coaches willing to use sports simulators for performance tracking. They might be lost in choosing the right sensors and analysis methods, or have doubts whether they can detect the aspects of performance that are relevant to them. Various sports have benefited from VR technologies as their interactivities overcome video playback drawbacks.^1^ Thanks to improvements in motion capture technology, tracking behavioral changes of players is possible without constraining their movements.^2^ VR technologies also contribute to the understanding and training of perception‐action coupling in sport.^1^, ^3^, ^4^, ^5^

Basketball free‐throw shooting is a perceptual‐motor task that can be perceived as simple, yet paradoxically misunderstood. On one hand, the time pressure to perform free‐throw is low, the thrower stands still, and the opponents cannot get between the player to stop the ball. On the other hand, experienced players' performance are extremely variable, ranging from 61.9% to 91.2% for the top 50 NBA players.^6^ In addition, up to 71.53% of the time, the winning team uses free‐throws,^7^ which account for 19 to 25% of the points in professional league games.^8^

### 1.1 The scales of analysis in basketball shooting

Depending on the systems and relevant aspects of performance, various scales of analysis can be used.^9^ At the scale of task performance, the success‐rate can reveal perception‐action mechanisms like the influence of floor marking,^10^, ^11^ changes in shooting distance,^10^, ^12^, ^13^, ^14^ and expertise.^12^, ^15^ At the scale of ball kinematics, an increase in shooting distance increases ball release speed,^14^, ^16^, ^17^, ^18^, ^19^ and decreases ball release angle.^14^, ^17^ The player's expertise seems to have no impact neither on the ball release speed nor on the ball release angle.^20^, ^21^ This phenomenon could be explained by the physics of basketball throwing. Various numerical simulators have shown that a combination of speed, angle, and rotation of the ball at release can lead to scoring.^22^, ^23^, ^24^, ^25^ However, the release parameters result in a large variance in the percentage of success as they may induce bounces before scoring. Therefore, differences in expertise can be observed in the ball‐to‐basket system but not in the ball‐to‐player system. Finally, at the scale of body kinematics, there is ample experimental evidence about the influence of distance, expertise, and gender. Concerning the influence of shooting distance, experienced players exhibit an increase of both shoulder^17^, ^26^ and elbow^14^, ^16^, ^17^, ^26^ angular velocities, an increased trunk tilt toward the basket,^16^, ^17^ an increased ankle and shoulder angles,^17^ a decrease of the elbow, trunk, knee, and shoulder angles^27^ with increasing basket distance. Few results describe the influence of expertise on players' kinematics. For example, Hudson^20^ observed a decrease in trunk rotation with expertise for young female players. Regarding the influence of gender, Vencúrik^18^ found that male players show higher shoulder angles at ball release, compared to their female counterparts.

### 1.2 Scales of analysis as indicators of simulator fidelity

Tracking basketball shooting in VR is challenging since variations of performance between players (different expertise or gender) and within the same players (at different shooting distances) are observable across the basket‐ball, ball‐player, and player systems. Coaches are looking for tools to detect the best players and follow their progress. Such feature is called *Construct Fidelity* and it depends on the ability of the simulator to distinguish real‐world experts from novices at the scale considered. Moreover, coaches need a basketball simulator that elicits realistic behaviors in all players at different scales.^28^ Meeting this so‐called *Ergonomic and Biomechanical Fidelity* is difficult as basketball simulators may lead to lower ball speed, as well as higher ball release height and landing angle, compared to the real environment.^29^

The second challenge is related to the extreme tuning of experienced players to the ball. The basketball is propelled into the basket with finely tuned finger movements. To meet the requirement of functional validity, the simulator must preserve such natural user‐interfaces.^5^, ^30^, ^31^ At the same time, the accuracy of experts' motor skills prohibit any simulation errors in basketball trajectory, as one‐degree error in the simulation of virtual ball trajectory may lead to unexpected bounces and not entering the rim.^32^ Therefore, to meet the *Physical Fidelity* requirements, a basketball simulator must animate the virtual ball's flight under normal laws of physics and bounce the virtual ball off the rim, backboard, and damp realistically.^4^, ^28^

The third challenge is related to the correct perception of the distance between the shooter and the basket.^33^ The simulator must provide the visual sources of information that allow adapting the throw to the basket distance. This information is carried either by the basket (e.g., the angle of elevation above the player's line of sight, motion parallax, and binocular vision^34^), or provided by the surrounding elements of the basketball court, including floor marking.^10^, ^11^ Therefore, to meet the *Psychological Fidelity*, a basketball simulator must emulate similar information pickup and in return, similar motor adjustments to the real basketball.^4^, ^28^

### 1.3 Aims of the study

The purpose of this study was to understand what a basketball simulator can reveal about shooting performance at different scales of analysis. We hypothesize that success‐rate, ball kinematics, and body kinematics can distinguish individual characteristics, thus certifying the construct validity of the simulator. Secondly, between‐player differences can mimic real‐life values, thus confirming the biomechanical fidelity of the simulator. Finally, by observing the within‐player differences as a function of basket distance, we hypothesize that players can perceive the distance to the basket and adjust their shots accordingly, thus demonstrating the psychological fidelity of the simulator.

## 2 METHODS

### 2.1 Population

Twelve experienced (3 females; age 21.0 ± 2.7 years; height 1.91 ± 0.19 m) and 10 novice (5 females; age 30.4 ± 5.5 years; height 1.74 ± 0.73 m) basketball players volunteered. Experienced participants played basketball at competitive levels ranging from departmental to professional national. Novice participants were recruited from the Faculty of Sport Sciences, Aix‐Marseille University, France, and reported recreational or no experience of playing basketball. All participants had normal or corrected‐to‐normal vision and were informed about the experimental procedures but not about the purpose of the study. Before the experiment, they provided written informed consent forms in accordance with the Declaration of Helsinki. The protocol was approved by the local ethics committee.

### 2.2 Apparatus

**FIGURE 1:**
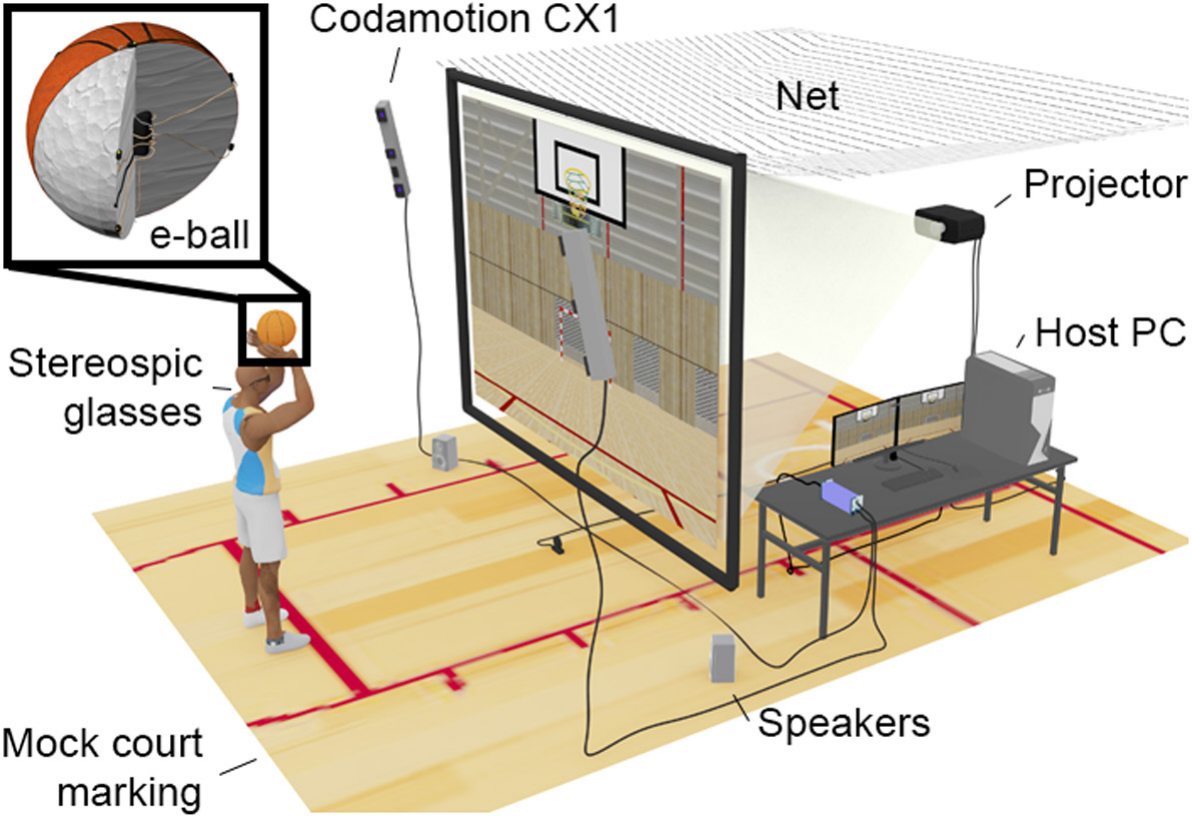
Arrangement of the hardware components of the basketball throwing simulator installed in a 6 m long × 5 m high room; the cross‐section depicts the structure of the instrumented ball with two Codamotion MarkerDriveBox embedded in Styrofoam and covered by a real basketball skin.

Basketball players naturally threw an instrumented ball, made with the leather texture of an official size 7 ball for men (TF1000, Spalding). The air bladder was replaced by an expanded polystyrene sphere inside which two MarkerDriveBoxes and their batteries (Charnwood Dynamics Ltd) were inserted. The eight active markers, two strobes, and two USB ports flushed the surface of the leather. The shape (0.6 mm variations of the surface height around the sphere), radius (11.84 cm), mass (630 grams), and appearance of the instrumented ball complied with FIBA regulations. A realistic virtual basketball court was stereoscopically back‐projected on a large screen (3.28 × 2.47 m, 1280 × 1024 pixels, at 60 Hz for each eye). Players observed the scene in 3D using stereoscopic glasses (Edge RF, Volfoni), synchronized by radiofrequency (ActivHub RF50, Volfoni), with the active stereoscopic projector (F35, Barco). The glasses were also equipped with a MarkerDriveBox connected to four active markers. The 3D translations and rotations of the ball and the glasses were measured by two optoelectronic units (Codamotion Cx1, Charnwood Dynamics Ltd) which sent the positions and rotations to the host computer running an OpenGL real‐time 3D engine.^35^ The software extrapolated the ball trajectory in real‐time, updated the virtual scene according to the player's point of view, and rendered the trajectory of the virtual ball on the screen when the ball passed behind the screen. Once thrown, the ball landed on the other side of the screen and was collected by the experimenters (Figure 1).

### 2.3 Procedures and independent variables

Before the experiment, each player performed a sequence of 10 warm‐up throws, followed by 10 extra throws in a real basketball court. They were then taken to the simulator room and instructed to behave as naturally as possible. They familiarized themselves with the simulator and the experimental procedure by performing 10 warm‐up throws. In the familiarization phase, participants were informed that the basket was located at the free‐throw distance. They also received feedback after each shot by watching their ball trajectory using an animated third‐person view.

**FIGURE 2:**
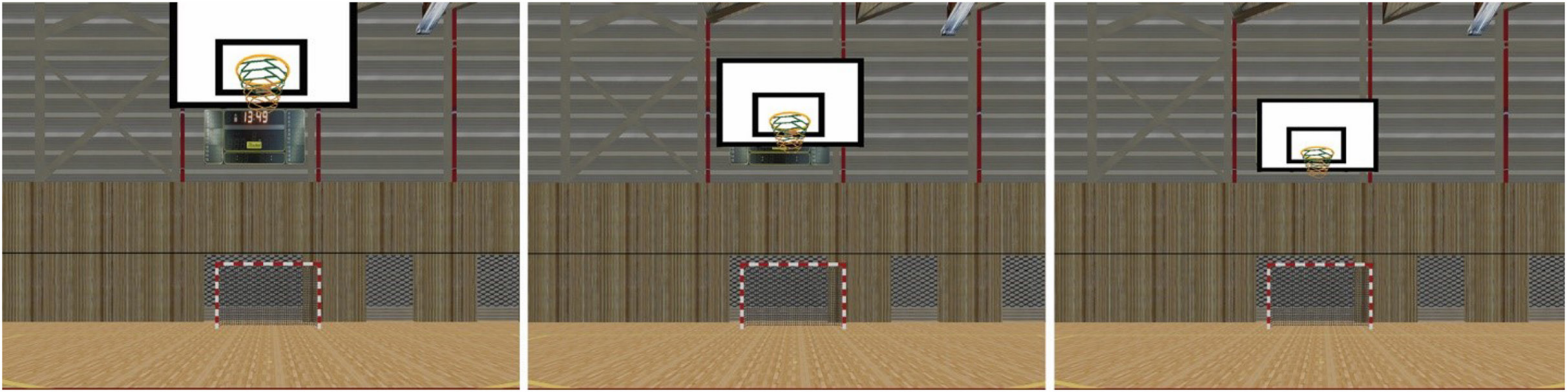
From left to right, screenshots of the visual appearance of the basket from the player's point of view at 3.225, 4.225, and 5.225 m from the free‐throw line. Note that the visual contextual information about the distance, carried by the surrounding virtual court, remained identical between trials, making them useless for perceiving basket distance and allowing to test the sufficiency of visual information about distance carried by the basket (e.g., backboard, hoop, and damp) to perform accurate throws.

During the experimental session, we manipulated the basket distance by displacing it in the virtual environment relative to the throwing point (3 conditions; 3.225, 4.225—the official distance between the center of the basket ring and the free‐throw line, and 5.225 m). The optical appearance of the basket in the three distances changed according to the real situations (Figure 2). Participants were never informed about the actual basket distance and were instructed to adjust their throw according to the perceived basket distance from trial to trial, and according to the visual information they picked up from the whole visual scene before each throw. No instructions were given on how to perform the shots (i.e., feet remaining on the ground or in the air) to prevent players from adapting their throw to the information relative to distance regulation. For each distance, participants threw the ball three times and in a random order.

### 2.4 Scales of analysis and dependent variables

The first scale of analysis of shooting performance examined the interaction between the virtual basket and the ball by computing the players' %success‐rate in VR. The second scale focused on the ball‐player system and through the measurement of the ball's speed and angle at the moment of release. These information were extracted from the virtual ball trajectories provided by the Codamotion system. Ball release was identified as the maximum speed reached by the ball before its apex. The third scale scrutinized players' body kinematics. Each participant was instrumented with 41 reflective markers (Qualisys Sports Marker Set, Qualisys AB, Sweden). The 3D position of each marker was simultaneously collected at 179 Hz using an eight‐camera Oqus 5 optical motion capture system (Qualisys AB) in a specific acquisition software (Qualisys Track Manager 2019, Qualisys AB). The start and stop of the shooting movements were identified a posteriori from 3D kinematics, when players raised the ball for the first time by flexing their elbow and at the ball release, respectively. From this time series, we computed the duration of the shooting movement. Then, we screened each throw to categorize the type of shooting movement: free‐throw when the participants' feet remained on the ground, and jump‐shot when they jumped during their shots. Finally, we used a 3D motion analysis package (Visual3D v6, C‐Motion) to compute wrist, elbow, shoulder, trunk, knee, and ankle joint angles, as well as release height and elbow and shoulder angular velocities at ball release.

### 2.5 Statistical analysis

For each dependent variable, the values obtained from the three shooting repetitions at each basket distance in the simulator were averaged. The influence of basket distance, expertise, and gender were analyzed using three‐way analysis of covariance (MANCOVA). Release height was set as the covariate.^17^, ^20^ To explore whether there is a relationship between type of throwing (free‐throw vs. jump‐shot) and basket distance (3.225, 4.225, and 5.225 m), expertise (experienced vs. novice), and gender (male vs. female), we used Pearson's chi‐square test. The level of significance was set to 0.05, and SPSS Statistics 27 (IBM) was used for all statistical analyses. Normality and homogeneity of variance were checked, and in the case of abnormal distribution and non‐homogeneity, alternative statistics were applied.

## 3 RESULTS

Multivariate tests showed that distance (*F*(24, 84) = 4.62, *p* < 0.001; Wilks' Λ = 0.19, $\eta_{p}^{2}$ = 0.57), expertise (*F*(12, 42) = 2.70, *p* = 0.009; Wilks' Λ = 0.56, $\eta_{p}^{2}$ = 0.43), gender (*F*(12, 42) = 3.92, *p* < 0.001; Wilks' Λ = 0.47, $\eta_{p}^{2}$ = 0.53), and interactions of expertise and gender (*F*(12, 42) = 3.75, *p* < 0.001; Wilks' Λ = 0.48, $\eta_{p}^{2}$ = 0.52) statistically significantly affected the combination of dependent variables when we controlled the ball release height. All numerical values of the dependent variables are reported in Table S1.

### 3.1 Scale of basket‐ball system

**FIGURE 3:**
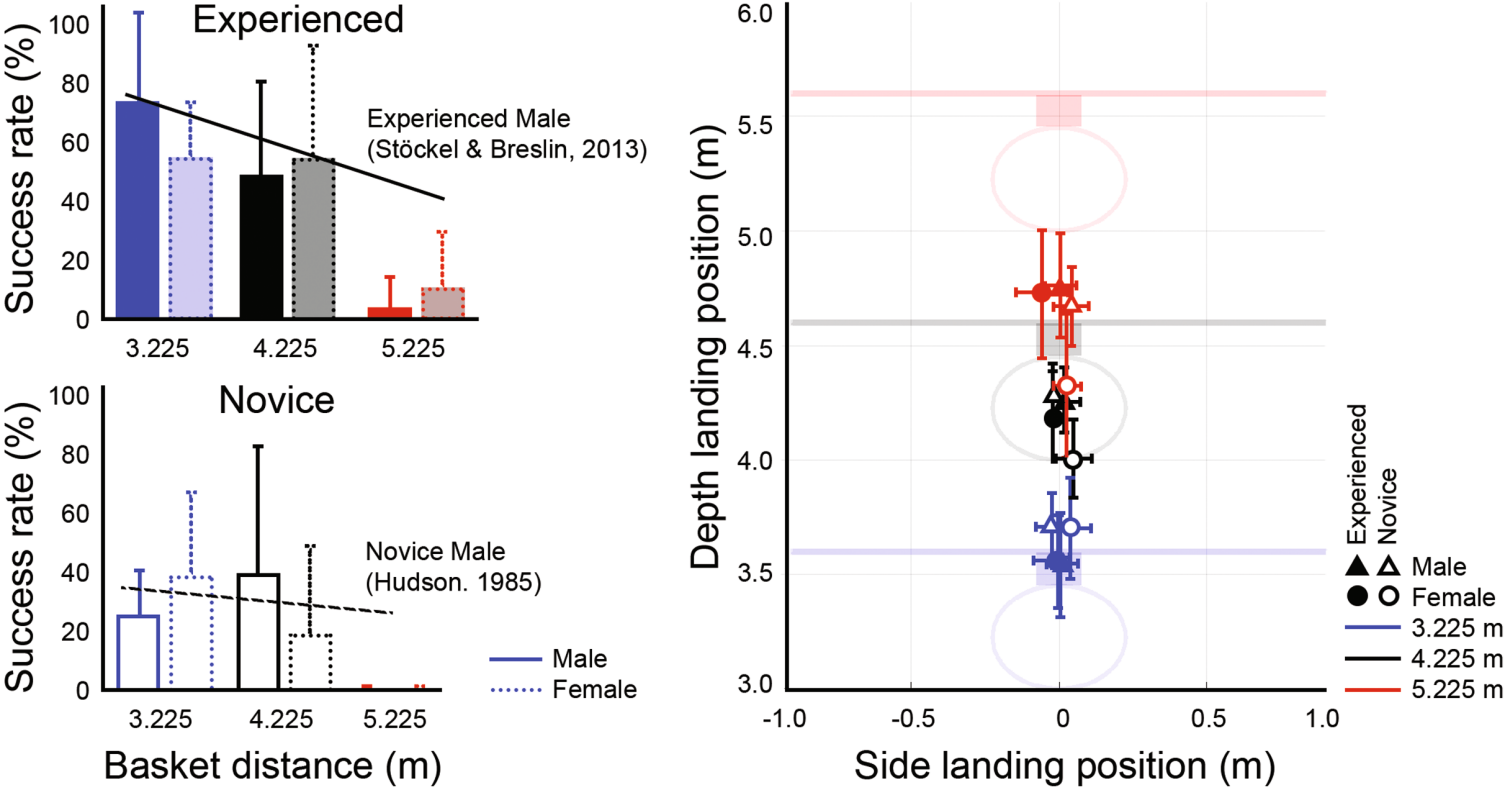
Interindividual average values of success‐rate, measured in the simulator, as functions of basket distance and gender, for experienced and novice players (left panel). The vertical bars depict the standard deviation of individual values. Previously reported success‐rate for comparable shooting distances in the real environments are shown with dotted lines. The right panel shows the top view of the interindividual mean values of ball landing depth positions plotted against basket distance. It confirms that the observed changes in success‐rate reflect a change in ball trajectory, and consistent with basket distance manipulation.

At free‐throw distance, experienced players had higher success‐rate compared to the novice players, both in the basketball court (65 ± 17 vs. 26 ± 19%) and within the simulator (50 ± 32 vs. 20 ± 36%). Regardless of the basket distance, experienced players also had higher success‐rate within the simulator and compared to the novice players (Figure 3; 41.2 ± 4.9 vs. 22.8 ± 5.5%; *F*(1, 53) = 6.37; *p* = 0.02; $\eta_{p}^{2}$ = 0.11). Figure 3 shows that regardless of players' expertise, the longer the basket distance, the lower success‐rate would be (*F*(2, 53) = 13.30; *p* < 0.001; $\eta_{p}^{2}$ = 0.33). Post hoc tests revealed that success‐rate was significantly higher at 3 m than 5 m (50.4 ± 6.0 vs. 8.3 ± 6.0%; *p* < 0.001) and at 4 m than 5 m (37.2 ± 6.0 vs. 8.3 ± 6.0%; *p* = 0.003).

### 3.2 Scale of ball‐player system

**FIGURE 4:**
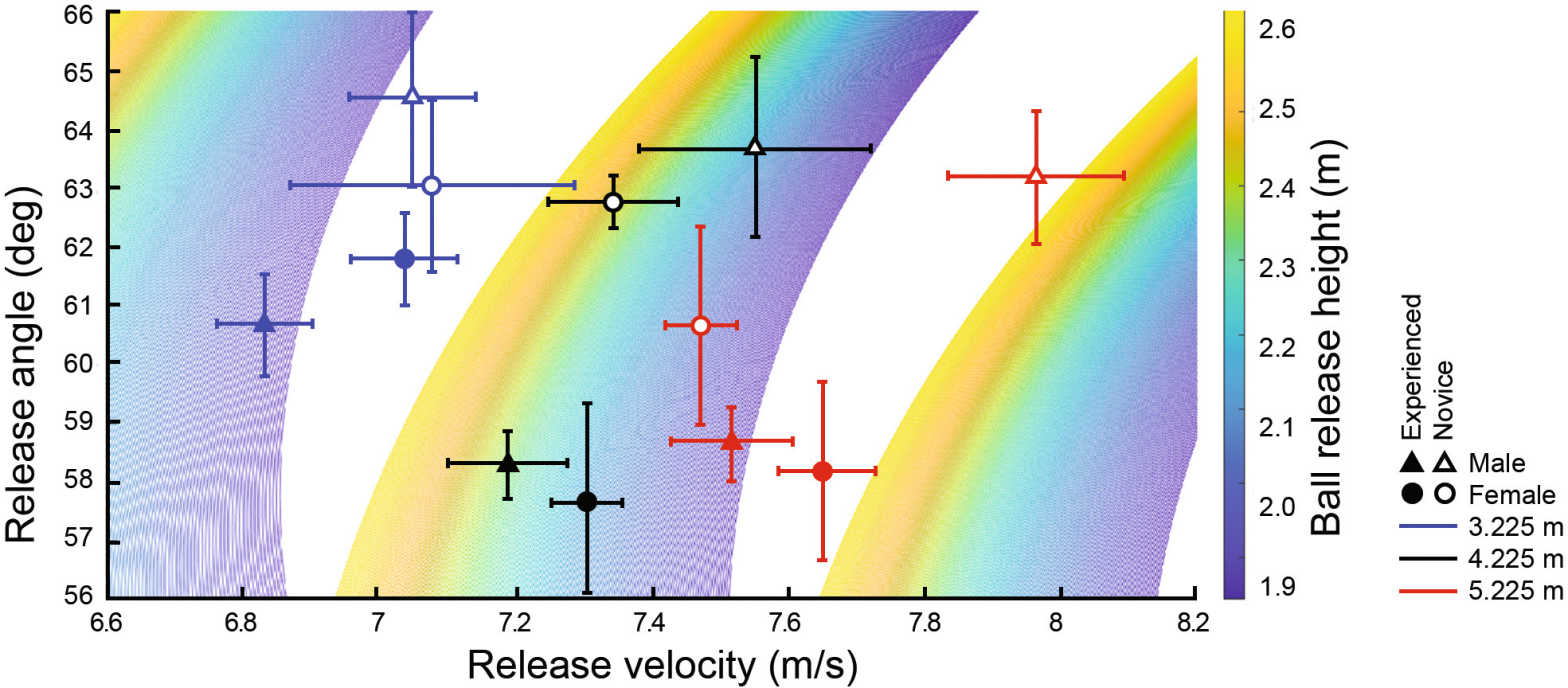
Relationship between interindividual values of the ball angle and velocity at release for experienced and novice players (plain and empty symbols, respectively), for male and female players (triangle and circle symbols, respectively), and at three basket distance (red, black, and blue, for 3.225, 4.225, and 5.225 m, respectively). The horizontal and vertical bars depict the standard errors of individual values. The colored areas represent the set of possible release parameters for swish scoring obtained with numerical simulation (see Gablonsky and Lang^22^) for each of the three different basket distances at a given release height (see the color bar). Scoring is still possible with higher ball velocity and different angles at release but conduces to backboard bounce.

Figure 4 shows that players modified the ball release velocity and angle as a function of basket distance. This was supported by a significant main effect of distance on ball release angle (*F*(2, 53) = 4.15; *p* = 0.02; $\eta_{p}^{2}$ = 0.14) and velocity (*F*(2, 53) = 37.15; *p* < 0.001; $\eta_{p}^{2}$ = 0.58). Post hoc tests showed that players released the ball at a higher angle when the basket was at 3 m compared to 5 m (62.5 ± 0.6 vs. 60.3 ± 0.6 deg, *p* = 0.03). Post hoc tests also showed that ball release velocity increased with basket distance (7.0 ± 0.1, 7.3 ± 0.1, and 7.6 ± 0.1 m/s for 3, 4, and 5 m basket distance, respectively; all *p* < 0.001). Test of between‐subjects effects also revealed that male players released the ball with a lower angle compared to the female players (59.7 ± 0.4 vs. 62.6 ± 0.6 deg; *F*(1, 53) = 11.73; *p* = 0.001; $\eta_{p}^{2}$ = 0.18). Although not statistically significant, novice players released the ball at a higher angle and at a greater speed, compared to the experienced players.

### 3.3 Scale of player system

Although not statistically significant between distances, players used more free‐throw style at shorter distances (14, 11, and 9 shots at 3, 4, and 5 m, respectively) and more jump‐shot style at longer distances (8, 11, and 13 shots at 3, 4, and 5 m, respectively; *p* > 0.05). Experienced players shot more with free‐throw style than their novice counterparts (*χ*[1] = 17.49, *p* < 0.001; Phi and Cramer's *V* = 0.52). Male participants shot more with free‐throw (27 vs. 7 shots) and fewer with jump‐shot style (15 vs. 17 shots) compared with female players (*χ*[1] = 7.54, *p* = 0.01; Phi and Cramer's *V* = 0.34).

**FIGURE 5:**
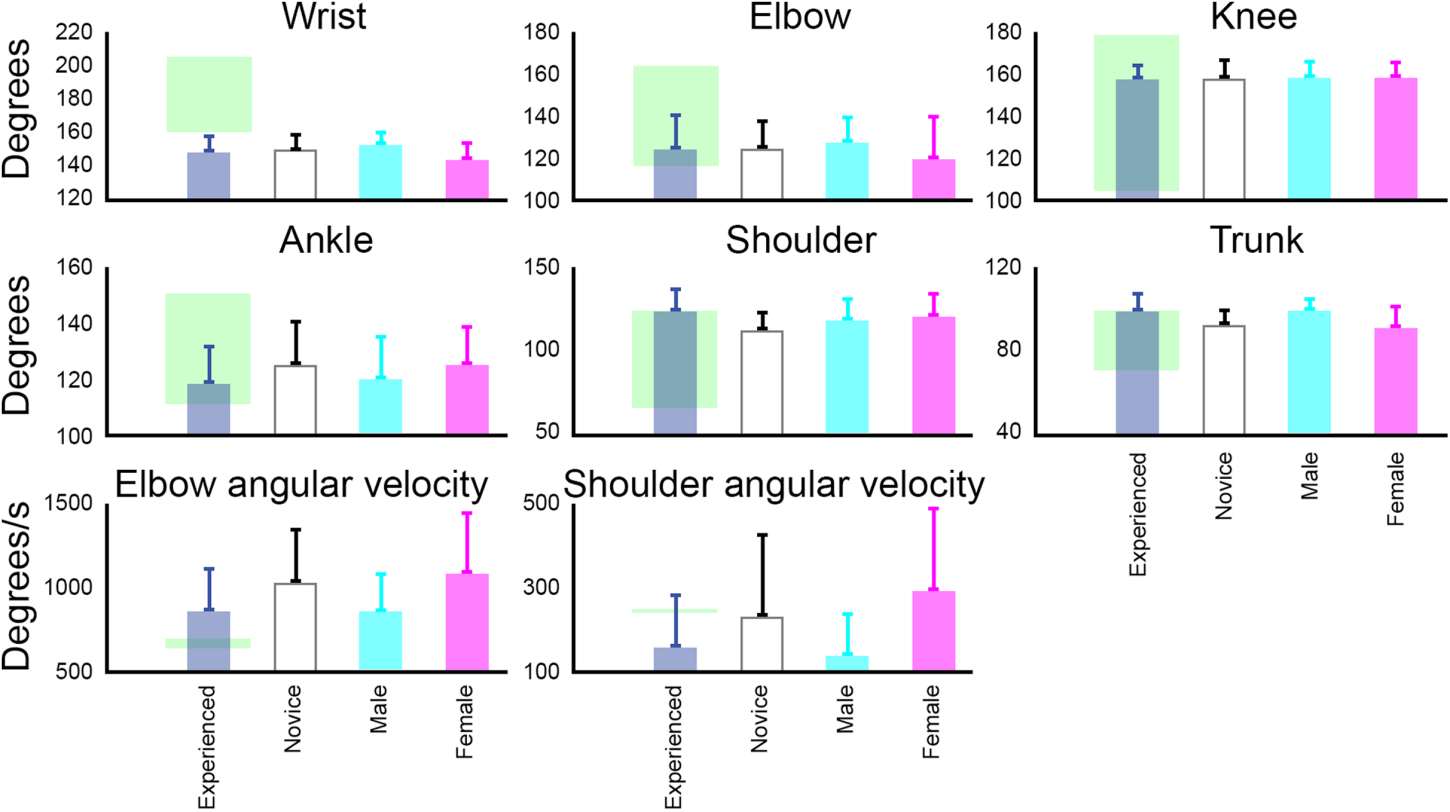
Interindividual average values of joint angles and angular velocities for experienced and novice players (with mixed gender) and for male and female players (with mixed expertise). The green areas depict the range of mean values reported at the free‐throw distance for each variable in the literature.^14^, ^16^, ^18^, ^36^, ^37^, ^38^, ^39^

Figure 5 shows interindividual average values of kinematic parameters that were influenced by the main effects of expertise and gender. Test of between‐subjects effects showed that experienced players had lower wrist (148.6 ± 9.4 vs. 150.4 ± 8.8 deg; *F*(1, 53) = 5.77; *p* = 0.02; $\eta_{p}^{2}$ = 0.10), and ankle angle (115.4 ± 11.2 vs. 121.5 ± 12.1 deg; *F*(1, 53) = 4.36; *p* = 0.04; $\eta_{p}^{2}$ = 0.08), as well as higher shoulder angle (122.8 ± 13.0 vs. 113.6 ± 9.9 deg; *F*(1, 53) = 7.24; *p* = 0.01; $\eta_{p}^{2}$ = 0.12) compared to novice players. Male players also exhibited lower shoulder angle (121.1 ± 12.5 vs. 127.9 ± 13.9 deg; *F*(1, 53) = 4.05; *p* = 0.04; $\eta_{p}^{2}$ = 0.07), higher wrist angle (151.9 ± 7.0 vs. 138.7 ± 9.1 deg; *F*(1, 53) = 8.26; *p* = 0.006; $\eta_{p}^{2}$ = 0.13), and lower shoulder angular velocity (118.1 ± 74.6 vs. 269.3 ± 96.4 deg/s; *F*(1, 53) = 5.51; *p* = 0.02; $\eta_{p}^{2}$ = 0.09) compared to female participants. Finally, the interactions of expertise and gender had statistically significant effects on movement duration (*F*(1, 53) = 5.75; *p* = 0.02; $\eta_{p}^{2}$ = 0.10), ankle angle (*F*(1, 53) = 10.29; *p* = 0.002; $\eta_{p}^{2}$ = 0.16), trunk rotation (*F*(1, 53) = 9.66; *p* = 0.003; $\eta_{p}^{2}$ = 0.15), and elbow angular velocity (*F*(1, 53) = 7.96; *p* = 0.007; $\eta_{p}^{2}$ = 0.13) at ball release moment.

## 4 DISCUSSION

In this study, we analyzed the shooting performance in VR at the three scales of basket‐ball, ball‐player, and player systems through measurement of success‐rate, and ball and body kinematics. Below, we discuss the results obtained at each scale of analysis with regards to the simulator's construct, biomechanical, and psychological fidelity.

### 4.1 Construct fidelity confirmed at three scales of analysis

Distinguishing the real‐world experienced and novice players is probably the most important feature of the simulators. This elicits the construct fidelity of the simulator.^4^, ^28^ At the scale of the basket‐ball system, our simulator allowed experienced players to exhibit higher success‐rate than novices. To the best of our knowledge, our simulator is the only one that can distinguish between novice and experienced players using success‐rate, as it is done in the real conditions.^12^, ^15^, ^20^

At the scale of ball‐player system, expertise did not influence ball release parameters, which was consistent with literature.^20^, ^21^ At the scale of the player system, several variables revealed that experienced players behave differently from novice players. First, experienced players mostly performed free‐throws, and showed lower ankle angle values compared to novice players. These observations are consistent with the literature which highlights that jump‐shots decrease stability in the ball release, and therefore, leads to less successful shots.^40^, ^41^

The ability of the simulator to distinguish between male and female players is perhaps less sought after. However, it plays a role in certifying our simulator's construct fidelity. At the scale of the basket‐ball system, our simulator was unable to detect gender. Although Liu and Burton^13^ reported higher success‐rate for novice males relative to their female counterparts, this might depend heavily on the sample tested. At the scale of the ball‐player system, male players released the ball at a lower velocity than females. The lower ball velocity allowed male players to perform an optimal ball trajectory,^25^ possibly due to higher ball release height compared to female players. Similarly, at the scale of player system, male players' lower shoulder angular velocity explains the lower ball release velocity. Likewise, male players mostly shot with free‐throw style, which suggests that they were not compensating the distance with faster propulsive movements by jumping. Taken together, these results suggest that individual differences can be tracked with our simulator at the three scales of analysis, thus validating its construct fidelity.

### 4.2 Biomechanical fidelity confirmed at three scales of analysis

Biomechanical fidelity ensures natural behavior in VR, and might allow the transfer from virtual training to real‐world situations. At the scale of the basket‐ball system, our simulator accurately reflected the free‐throw success‐rate of the players compared with their performance measured in real‐world conditions and were similar to those observed by Hudson^42^ and Button et al.^36^ Moreover, success‐rate decreased with the increase of basket distance as reported in real‐conditions for both novice^12^, ^13^ and experienced players.^10^, ^12^, ^14^

However, to fully attest the biomechanical fidelity of our simulator, the “especial skill” performance^10^, ^11^, ^43^ seems to be missing. The “especial skill” performance is a higher shooting accuracy observed in experienced players at free‐throw distance compared to the closer basket distances. The hypothesis of lack of expertise of our participants, for explaining the absence of this phenomenon can be ruled out. Indeed, our participants included seven athletes playing in the first two national leagues, two playing at the regional level, and four at the departmental level. We suggest that the neutralization of the visual context in our experimentation, that is unique to the free‐throw line for highlighting the contribution of the basket visual information, is responsible for the absence of this phenomenon. It is therefore possible that the “special skill” can be observed with our simulator, if the distance to the basket is naturally manipulated by moving the surrounding field and the basket. It is also possible that additional methodological precautions are needed to observe the performance of the “special skill.” In our study, the participants performed only a few shooting repetitions at each distance (three for each distance) compared with 30,^10^ 40,^11^ and 50^43^ repetitions at each distance in the literature.

At the scale of the ball‐player system, comparison of ball velocity and angle with numerical simulations showed that the obtained data were consistent with optimal ball‐release parameters,^22^, ^23^, ^24^, ^25^, ^44^ and marks the difference with previous shooting simulation attempt.^29^ One might ask about the adoption of optimal parameters by both novice and experienced players. The lack of differences between expert and novice players in ball release parameters were already evidenced.^42^ This result can be explained with numerical simulations, which attests that several combinations of ball speed, angle, and rotation can lead to successful shots. Although these differences are not significant, novice players released the ball at a slightly higher angle than the experienced players.^42^ This suggests that novices exploited marginal parameters among the optimal ones compared to experienced players. At the scale of the player system, the analysis of kinematics showed that all joint angles and angular velocities fell within the ranges of previously reported data in real basketball.^14^, ^16^, ^18^, ^36^, ^37^, ^38^, ^39^ In sum, our results were comparable with real‐word scenarios, and validate the biomechanical fidelity of our simulator.

### 4.3 Psychological fidelity confirmed at two scales of analysis

Only two scales of analysis allowed tracking shooting adaptation to the basket distance. Similar to the real conditions and at the scale of basket‐ball system, success‐rate was noticeably decreased when the distance was increased.^10^, ^12^, ^13^, ^14^ At the scale of ball‐player system, ball release speed increased with shooting distance,^14^, ^16^, ^17^, ^45^ while the release angle consistently decreased.^14^, ^16^, ^17^ The player system scale only allowed tracking the shooting adjustments, in response to the manipulation of basket distance, and through shooting style. Shooting style switched from free‐throw at short basket distance to jump‐shot at the largest basket distance. This was consistent with the biomechanical requirements for increasing the release height with higher distances^46^ and similar to the previous literature.^13^, ^37^

Overall, several body kinematics variables changed consistently with the increase of distance, but without any statistical significance. Joint angles at release moment were influenced by expertise and gender, but not by the basket distance. Nevertheless, we noticed that some variables evolved, though not significantly, in a way consistent with the distance manipulations. Players' trunks rotated toward the basket,^16^, ^37^ ankle angle increased,^14^, ^16^ and elbow and shoulder angular velocities increased with the increase of shooting distance.^17^ Perhaps advanced mathematical descriptions such as uncontrolled manifold or principal component analysis (see Ibáñez‐Gijón et al,^9^ for an implementation in basketball) would help to understand how whole‐body kinematics are controlled during distance adaptation. However, the cost and complexity of such procedures to coaches are questionable when ball‐basket and play‐ball scales offer sufficient sensitivity. Therefore, the psychological fidelity of our simulator was attested by changes at ball‐player system scale that cascaded on the basket‐ball scale. Changes at the player system scale must have occurred for changing the ball release parameters as a function of perceived distance from the basket. The ball release parameters are functionally the outcome of body movements but angular measurements may not be sufficient to capture these kinematic changes. This is probably because changes in joint angles cannot be assessed independently, but in synergy with other joints to propel the ball significantly differently, and to cope with changing basket distances.

This study showed that VR could be an appropriate tool for investigating the role of contextual pieces of information about distance in basketball shooting. Since neither the free‐throw line nor the virtual background changed with basket distance manipulation, looking at the backboard and the rim provides sufficient visual information about the basket distance, and allow novice and experienced players to regulate their throws. Our results are therefore consistent with gaze‐tracking studies, suggesting that players' gaze at the basket just before and during shooting,^47^, ^48^, ^49^ as well as the availability of basket vision during the final part of the shooting movement^50^, ^51^ play functional roles in the information pick‐up. This also questions the role of visual information sources provided by the floor marking^10^ and promotes the rim and basket as primary (or even sole) sources of perceptual input for the players.^43^

### 4.4 Practical implications

The practical implications are two‐folded for trainers. First, our results can advise on appropriate and affordable equipment. Second, our results can allow coaches to select scenarios that benefit their training session. Concerning the selection of equipment, it is important to keep in mind that in basketball shooting, success‐rate, and the ball and body kinematics form a continuum in the complexity of levels of analysis of performance. They are also featured by the material needs of increasing complexity. We present here different hardware alternatives, that are doable in VR, to access them. Coaches interested in success‐rate measurement should resort to commercial wearables, sensors integrated into a ball, or smartphones that capture and deliver shooting outcome analytics. Ball kinematics are also important to monitor, given the efforts made by physicists to determine optimal release conditions and their relationship with the probability of successful throws. Coaches willing to monitor ball kinematics can benefit from commercial ball‐embodied sensors. Coaches attempting to optimize players' shots should also pay attention to systems that allow measuring ball backspin.^25^ Finally, body kinematics analysis implies using more complex and expensive systems that rely on optoelectronics or inertial units. The accuracy of optoelectronic systems surpasses inertial ones but are less portable and require more effort for installation and calibration. The choice of an appropriate marker set is critical and ensures that all of the required variables can be calculated from the collected raw data. Subject preparation and careful placement of markers are other issues that need attention. Finally, smart choices can be made for the measurement of certain kinematics‐dependent variables. For example, foot switches are more easily implemented than measuring ankle angle to distinguish between free‐throw from jump‐shots.

Regarding the scenarios, the basic use of such basketball shooting simulator is reliable enough to allow coaches to track the progress of players during injury recovery and to compare them to a database to certify their levels during the off‐season. Other more complex scenarios can also allow detecting the perceptive expertise of players more precisely. In the same way that contextual information was neutralized using VR in this paper, other manipulations are possible including spatial^34^ and temporal occlusion,^50^, ^51^ as well as decorrelation^52^ of the several sources of information available in the visual scene. These manipulations could be utilized to infer the contribution of visual information to the regulation and for regulating the motion. Learning protocols can also be defined to help novice players to better attune with relevant visual sources of information. Finally, dual situations can be defined with virtual opponents to detect and train players' ability to avoid the defensive actions of the opposing players.

### 4.5 Limitations

The present study propels VR as an effective tool for basketball coaches. Nevertheless, the conclusions drawn must be acknowledged by the following experimental limitations. Concerning the protocol to probe the ergonomic and psychological fidelities, our approach was based on comparisons of values acquired in the simulator with those from the existing literature. We appreciate that this protocol might be less powerful than a real vs. virtual comparison with the same players, as used in studies such as Harris et al^53^ and Vine et al.^54^ Concerning the sample, we had an unequal gender distribution in each group due to snowball sampling and the inclusion criteria. The generalization of the results could thus be further improved by using a more balanced sampling pool. Finally, a larger number of shooting repetitions will allow analyze stability, and perhaps allow revealing additional phenomenon like “especial skill” performance.

## 5 CONCLUSIONS

Coaches can trust the basketball shooting simulator to enable realistic behavior, to track between‐player differences, and to challenge the players' perceptual‐motor systems naturally. Coaches must be aware that the three scales of analysis of basketball shooting performance are not equally useful. Success‐rate accounts for players' adjustments to basket distance and allows tracking expertise. Ball kinematics also reflect the players' adjustments to basket distance and allow detecting gender but not expertise. Players' kinematics do not echo players' adjustments to distance but allow tracking their expertise and gender. Depending on specific aims, material expenses should also be considered.

## 6 PERSPECTIVE

Virtual reality could be used for monitoring the performance and progress of athletes. By using various sources of information from the environment, the ball, and the player, we explored the use of technology for detecting expertise. Our results also advance the research on visual perception in sport by demonstrating that virtual reality is an appropriate tool to study the role of visual information in distance perception. We also provided practical implications on what measurement devices could be used according to each level of analysis.

## CONFLICT OF INTEREST

The authors report there are no competing interests to declare.

## Supporting information


TableS1

## Data Availability

The data that support the findings of this study are available from the corresponding author upon reasonable request.
