# Supplementary material for: A multi‐scale analysis of basketball throw in virtual reality for tracking perceptual‐motor expertise
Source: Scand J Med Sci Sports. 2022 Nov 18;33(2):178–88. doi: 10.1111/sms.14250 (PMC10100508; doi:10.1111/sms.14250)
Supplement: Supplementary file 1 — TableS1 [file SMS-33-178-s001.docx]

Table S1. Mean ± SD of dependent variables.

| Dependent variables | Expertise | Gender | Basket distance (m) | | |
| --- | --- | --- | --- | --- | --- |
|  |  |  | 3.225 (free-throw - 1m) | 4.225 m (Free-throw) | 5.225 (free-throw + 1m) |
| Success rate (%) | E | M | 69.64±9.44 | 43.87±9.39 | -0.88±9.49 |
|  |  | F | 59.57±15.27 | 60.12±15.40 | 14.83±15.21 |
|  | N | M | 28.21±11.58 | 40.82±11.52 | 1.71±11.60 |
|  |  | F | 44.10±12.07 | 4.20±12.09 | 17.47±12.08 |
| Ball release angle (degrees) | E | M | 60.97±2.73 | 59.11±1.99 | 58.54±1.58 |
|  |  | F | 64.6±2.52 | 63.26±1.89 | 63.63±3.06 |
|  | N | M | 61.94±1.69 | 58.52±3.26 | 59.18±3.26 |
|  |  | F | 63.16±3.16 | 60.88±3.60 | 62.48±3.50 |
| Ball velocity at release (m/s) | E | M | 6.82±0.23 | 7.49±0.29 | 7.02±0.33 |
|  |  | F | 7.04±0.16 | 7.97±0.22 | 7.51±0.13 |
|  | N | M | 7.03±0.18 | 7.65±0.16 | 7.20±0.21 |
|  |  | F | 7.07±0.47 | 7.47±0.12 | 7.22±0.21 |
| Shooting type (FT and JS) | E | M | 8 and 1 (67% and 8%) | 7 and 2 (58% and 17%) | 5 and 4 (42% and 33%) |
|  |  | F | 3 and 0 (25% and 0%) | 2 and 1 (17% and 8%) | 2 and 1 (17% and 8%) |
|  | N | M | 3 and 2 (30% and 20%) | 2 and 3 (20% and 30%) | 2 and 3 (20% and 30%) |
|  |  | F | 0 and 5 (0% and 50%) | 0 and 5 (0% and 50%) | 0 and 5 (0% and 50%) |
| Movement duration (s) | E | M | 1.05±0.2 | 1.26±0.59 | 1.06±0.21 |
|  |  | F | 1.32±0.43 | 1.08±0.38 | 0.98±0.45 |
|  | N | M | 1.23±0.48 | 1.22±0.54 | 1.04±0.26 |
|  |  | F | 1.38±0.59 | 1.14±0.43 | 1.12±0.47 |
| Hand angle at ball release (degrees) | E | M | 151.61±5.61 | 151.28±8.55 | 152.85±7.36 |
|  |  | F | 138.49±11.41 | 140.05±8.91 | 137.52±10.87 |
|  | N | M | 148.33±9.04 | 148.47±9.66 | 149.02±10.44 |
|  |  | F | 152.46±9.73 | 151.97±11 | 155.13±7.71 |
| Elbow angle at ball release (degrees) | E | M | 125.47±10.69 | 124.83±9.77 | 126.83±8.61 |
|  |  | F | 110.47±17.82 | 118.38±20.19 | 130.72±8.62 |
|  | N | M | 121.72±13.67 | 122.48±14.32 | 128.77±8.38 |
|  |  | F | 123.67±8.9 | 126.97±14.65 | 126.92±12.45 |
| Shoulder angle at ball release (degrees) | E | M | 119.78±12.73 | 121.78±13.19 | 121.89±12.93 |
|  |  | F | 128±15.59 | 129.33±12.06 | 126.33±19.43 |
|  | N | M | 116.4±12.9 | 107.4±10.45 | 112±7.28 |
|  |  | F | 114.2±9.31 | 115.6±10.31 | 116.2±10.83 |
| Trunk rotation at ball release (degrees) | E | M | 97.11±3.14 | 96.11±3.02 | 95.67±2.96 |
|  |  | F | 86±8.19 | 86.17±7.29 | 85±8.54 |
|  | N | M | 90.2±3.11 | 90.4±4.22 | 90.8±2.17 |
|  |  | F | 89.8±6.91 | 89.8±4.55 | 91.4±6.88 |
| Knee angle at ball release (degrees) | E | M | 159.41±7.6 | 161.21±6.74 | 159.99±6.87 |
|  |  | F | 154.68±6.72 | 159.47±8.12 | 157.43±5.88 |
|  | N | M | 158.23±7.4 | 158.81±8.1 | 159.35±6.48 |
|  |  | F | 157.74±9.77 | 158.76±7.09 | 159.74±9.46 |
| Foot angle at ball release (degrees) | E | M | 113.09±12.68 | 116.87±12.28 | 121.29±10.71 |
|  |  | F | 107.05±8.81 | 109.5±4.32 | 114.44±6.19 |
|  | N | M | 111.58±11.77 | 115.03±11.14 | 119.58±10 |
|  |  | F | 113.56±15.06 | 116.24±14.3 | 117.46±14.66 |
| Elbow angular velocity at ball release (degrees /s) | E | M | 770.76±191.42 | 869.98±233.60 | 933.79±205.73 |
|  |  | F | 912.98±360.10 | 1010.71±113.65 | 847.25±580.26 |
|  | N | M | 837.74±236.86 | 947.6±296.57 | 907.9±239.98 |
|  |  | F | 1111.65±397.83 | 1264.57±315.03 | 1229.58±277.8 |
| Shoulder angular velocity at ball release (degrees /s) | E | M | 122.01±84.17 | 109.76±91.33 | 122.7±50.61 |
|  |  | F | 267.75±97.03 | 301.01±58.51 | 239.28±146.54 |
|  | N | M | 149.87±114.24 | 186.21±94.34 | 174.38±92.11 |
|  |  | F | 246.95±235.31 | 320±183.95 | 257.2±216.6 |

E: Expert; N: Novice; M: Male; F: Female; FT: Free throw; JS: Jump shot
